# Endogenous Retrovirus‐Like Particle‐Deficient CHO Cells Can be Generated by CRISPR or shRNA and Enriched Based on Cell‐Surface Expression of Retroviral Envelope Protein

**DOI:** 10.1002/bit.70043

**Published:** 2025-08-22

**Authors:** Matthew Stuible, Sergio P. Alpuche‐Lazcano, Christian Gervais, Manon Ouimet, Julie Lippens, Martine Pagé, Audrey Morasse, Anna N. Moraitis, Yves Durocher

**Affiliations:** ^1^ National Research Council Canada, Human Health Therapeutics Research Centre Montreal Quebec Canada; ^2^ Current affiliation: Departamento de Medicina Molecular y Bioprocesos, Instituto de Biotecnología Universidad Nacional Autónoma de México (UNAM) Cuernavaca Morelos CP 62210 México

**Keywords:** biomanufacturing, cell engineering, CHO cells, CRISPR, endogenous retrovirus‐like particles

## Abstract

Despite evidence that they are not functional or infective, retrovirus‐like particles (RVLPs), originating from endogenous proviral sequences in Chinese hamster ovary (CHO) cells, present a safety risk for biotherapeutics manufactured using this cell line due to their resemblance to other mammalian leukemia viruses. Here, we demonstrate that CRISPR‐ and shRNA‐based cell engineering strategies can be used to disrupt RVLP production by targeting the RVLP nucleotide sequences. Additionally, specific antibodies were generated to monitor RVLP protein expression, including RVLP envelope (Env) protein localized on the surface of CHO cells, greatly facilitating selection of RVLP‐deficient clones. These modified CHO cells showed reduced RVLP production while maintaining or enhancing the ability to produce recombinant virus‐like particles (VLPs), highlighting their potential application in biomanufacturing, especially for complex biologics that are incompatible with standard RVLP mitigation procedures, namely viral inactivation and nanofiltration.

## 1 Introduction

Like cell lines derived from mice and other mammals, Chinese hamster ovary (CHO) cells produce retrovirus‐like particles (RVLPs) resulting from endogenous proviral sequences present in their genomes. These sequences were not introduced as a result of infection by adventitious agents, but rather originate in the animals from which the cells were derived (Stocking and Kozak 2008; Duroy et al. 2020). Initial characterization of CHO RVLPs was reported over 50 years ago, including direct visualization of virus particle budding from the cell membrane by electron microscopy and supporting evidence including detection of reverse transcriptase activity and a retroviral antigen by ELISA in CHO culture media (Lieber et al. 1973). Through the 1980s and 90s, as CHO cells began to be used for manufacturing of recombinant protein therapeutics, CHO RVLPs were further characterized in several published studies. Importantly, even after evaluation by a number of groups, no evidence has been reported demonstrating that these RVLPs are capable of infection of any cell type in vitro. Furthermore, initial sequencing of RVLP proviral sequences from the CHO genome showed that open reading frames encoding retroviral proteins (Gag, Pol, and Env) were disrupted by mutations that would render them nonfunctional (Anderson et al. 1991; Lie et al. 1994), supporting their characterization as nonfunctional retrovirus‐*like* particles.

Despite these initial sequencing results, it remained possible that other yet‐to‐be‐sequenced RVLP proviruses could encode functional proteins, especially given the evidence that as many as 300 copies of homologous sequences could be present in the CHO cell genome (Anderson et al. 1991) and that RVLP budding from CHO cells, which would require functional Gag protein at a minimum, had been well‐established (Lieber et al. 1973). CHO cell genome assemblies published in the early 2010s were based on short‐read sequencing and lacked coverage of repetitive elements like RVLP proviruses. It was not until the first genome assembly based on long‐read sequencing in 2018 (Rupp et al. 2018) that the locations and sequences of individual proviruses could be characterized. A study by Duroy et al. (2020) focused specifically on genomic analysis of CHO RVLPs and how they could be disrupted using CRISPR, based on analyses by long‐read sequencing and other methods of the CHO‐K1 cell line (Duroy et al. 2020). Their results confirmed that > 100 RVLP provirus sequences are found in these cells, but two additional discoveries were unexpected: a large number of these proviruses contain intact Gag, Pol, and Env open reading frames, and more surprisingly, only a single integrated provirus sequence appears responsible for the great majority of RVLP production. This locus, named ETC109F, could be targeted with CRISPR to generate RVLP‐deficient CHO clones, supporting the feasibility of cell engineering by CRISPR or other methods to improve CHO clones by decreasing RVLP production.

Given the potential of RVLP‐deficient cells to facilitate therapeutic protein bioprocessing and also enable the use of CHO cells for recombinant enveloped virus‐like particle (VLP) production (Alpuche‐Lazcano et al. 2023; Sanchez‐Martinez et al. 2024a), we set out to characterize and disrupt RVLP production in CHO‐DXB11‐derived cell lines. In addition to elucidating the sequence of RVLP transcripts expressed by these cells and targeting them by CRISPR or shRNA, we also generated antibody reagents to monitor RVLP protein expression by western blot analysis and flow cytometry. We discovered that RVLP Env protein is readily detectable on the surface of these cells and that the absence of Env is an excellent marker for RVLP‐deficient cells. Finally, by Env‐based enrichment, we identified clones resulting from CRISPR or shRNA targeting which exhibit greatly reduced RVLP production but can produce similar or higher titers of recombinant SARS‐CoV‐2 spike‐based enveloped VLPs than unmodified parental cells.

## 2 Methods

### 2.1 Cells and Cell Growth Conditions

CHO^2353^ cells (Joubert et al. 2023) as well as derived RVLP knockout/knockdown clones were maintained in suspension culture in chemically defined proprietary media formulation as described previously (Alpuche‐Lazcano et al. 2023). Transfections and productions were performed using the same media. Cells were cultured in polycarbonate Erlenmeyer flasks with a 0.2 µm vent cap (Corning) under constant orbital shaking at 120 rpm in a humidified incubator at 37°C with 5% CO₂.

### 2.2 RNA Extraction, RT‐PCR, Cloning, and Sequencing

Total cellular RNA was extracted from 1 × 10^6^ cells using the RNeasy Mini Kit (Qiagen). To remove residual genomic DNA, an in‐solution digestion with DNase I followed by an additional RNA clean‐up step was performed as described in the RNeasy kit instructions. cDNA was generated using the QuantiTect Reverse Transcription kit using Oligo d(T)18 primers (New England Biolabs), with a control reaction performed without reverse transcriptase enzyme (to confirm absence of contaminating genomic DNA). PCR with cDNA template (RT‐PCR) was performed using Q5 polymerase (New England Biolabs) in a volume of 20 µL containing 1 µL cDNA, 0.2 µL Q5 enzyme, 0.5 µM forward and reverse primers, 0.2 mM dNTPs, and 1× Q5 reaction buffer. PCR products were purified using the Qiaquick PCR Purification Kit (Qiagen) and cloned into the pJet1.2 plasmid using the CloneJet PCR Cloning Kit (Thermo Fisher Scientific). Cloned PCR products were sequenced by Sanger sequencing using pJet1.2 forward and reverse sequencing primers.

### 2.3 SDS‐PAGE and Western Blot Analysis

SDS‐PAGE was performed using NuPAGE 4%–12% Bis‐Tris gels (Invitrogen). Protein samples were heat‐denatured at 70°C for 10 min under reducing conditions before separation by SDS‐PAGE. Total protein staining (Coomassie Blue) and western blot analysis were performed using standard methods. Cell lysates were prepared using mRIPA buffer (150 mm NaCl, 50 mm Tris‐HCl, pH 7.4, 1% Nonidet P‐40, 0.25% sodium deoxycholate) containing 1× Complete protease inhibitors (Roche). Lysates were cleared by centrifugation (1 min, 13,000*g*). Protein concentrations were determined by BCA assay to normalize quantities loaded on SDS‐PAGE gels. Antibodies used for western blot analysis were developed as described in the current manuscript, except for anti‐actin (catalog #A‐2066; Sigma) and anti‐spike S2A4 VHH‐human‐Fc (Rossotti et al. 2022).

### 2.4 Monoclonal Antibody Generation and Screening

Six‐week‐old female A/J mice (The Jackson Laboratory, Bar Harbor, ME) were immunized intraperitoneally and subcutaneously with 40 µg of a pool of Gag and Env antigens, based on CHO RVLP sequences, emulsified in TiterMax adjuvant (Cedarlane, Burlington, ON). A boost was administered on Day 21 with 75 µg of the same antigen pool diluted in PBS. A final boost of 75 µg of the pool was given to two mice on Days 246 and 256, 4 and 3 days before fusion, respectively.

Spleen cells from each mouse were harvested in Iscove's Modified Dulbecco's Medium (IMDM) and fused with the NS0 myeloma cell line at a 1:1 ratio using an electrofusion apparatus (ECM 2001, BTX; Harvard Apparatus, Holliston, MA) according to the manufacturer's instructions. After overnight incubation in selection medium (IMDM supplemented with 20% heat‐inactivated [H‐I] FBS, 1× hypoxanthine‐aminopterin‐thymidine [HAT] supplement, 1 ng/mL mouse IL‐6, 100 IU/mL penicillin, and 100 µg/mL streptomycin), the freshly fused hybridomas were washed and suspended at a concentration of 2–5 × 10⁵ input myeloma cells per mL in semi‐solid ClonaCell‐HY hybridoma selection medium D (StemCell Technologies, Vancouver, BC, Canada), supplemented with 5% H‐I FBS, 1 ng/mL mouse IL‐6, and 10 µg/mL fluorescein isothiocyanate (FITC)‐labeled F(ab′)_2_ goat anti‐mouse IgG (Jackson ImmunoResearch, Cedarlane, Burlington, ON). The cell suspension was plated in Nunc OmniTrays (Thermo Scientific) and incubated for 6 days at 37°C, 5% CO₂. A robotized mammalian cell clone picker (Clonepix2; Molecular Devices, Boston, MA) was used to pick and transfer fluorescent antibody‐secretor hybridoma clones into sterile 96‐well plates containing 200 µL of selection medium, with the HAT supplement replaced by the hypoxanthine‐thymidine (HT) supplement. Picked clones were further incubated for 3 days at 37°C, 5% CO₂.

Hybridoma supernatants from two fusion experiments were screened for antigen specificity by enzyme‐linked immunosorbent assay (ELISA) using the immunogens (data not shown) and those showing binding were further screened by flow cytometry using wild‐type CHO^2353^ and Env‐KO CHO‐C9 cells to identify clones with specific affinity for detecting Env at the cell surface. Briefly, after centrifugation, the cells were stained with Fixable Viability Stain 450 (FVS450, BD cat#562247) according to the manufacturer's instructions to exclude dead cells from the analysis. The cells were then resuspended in RPMI‐1640 medium supplemented with 10% heat‐inactivated FBS) at a cell density of 2 × 10⁶ cells/mL. All incubations were performed at 4°C. Fifty (50) µL/well of cells were added into a 96‐well plate (Costar #3363), and an equal volume of hybridoma supernatant was added and incubated for 2 h. The cells were washed twice by centrifugation and further incubated with an Alexa Fluor 647 (AF647)‐labeled F(ab′)_2_ goat anti‐mouse antibody (Jackson ImmunoResearch, Cedarlane, Burlington, ON) for 1 h. Samples were filtered through a 30‐40 µm mesh filter plate (Pall #PN8027) to remove cell aggregates. Flow cytometry analysis was performed on 2000 viable single‐cell events gated on forward scatter, side scatter, and FVS450 dye exclusion using a BD‐LSR Fortessa flow cytometer (Becton Dickinson Biosciences, CA, USA) and a standard filter set with BD FACSDiva acquisition software, according to the manufacturer's instructions.

### 2.5 CRISPR Targeting

**Figure 1:**
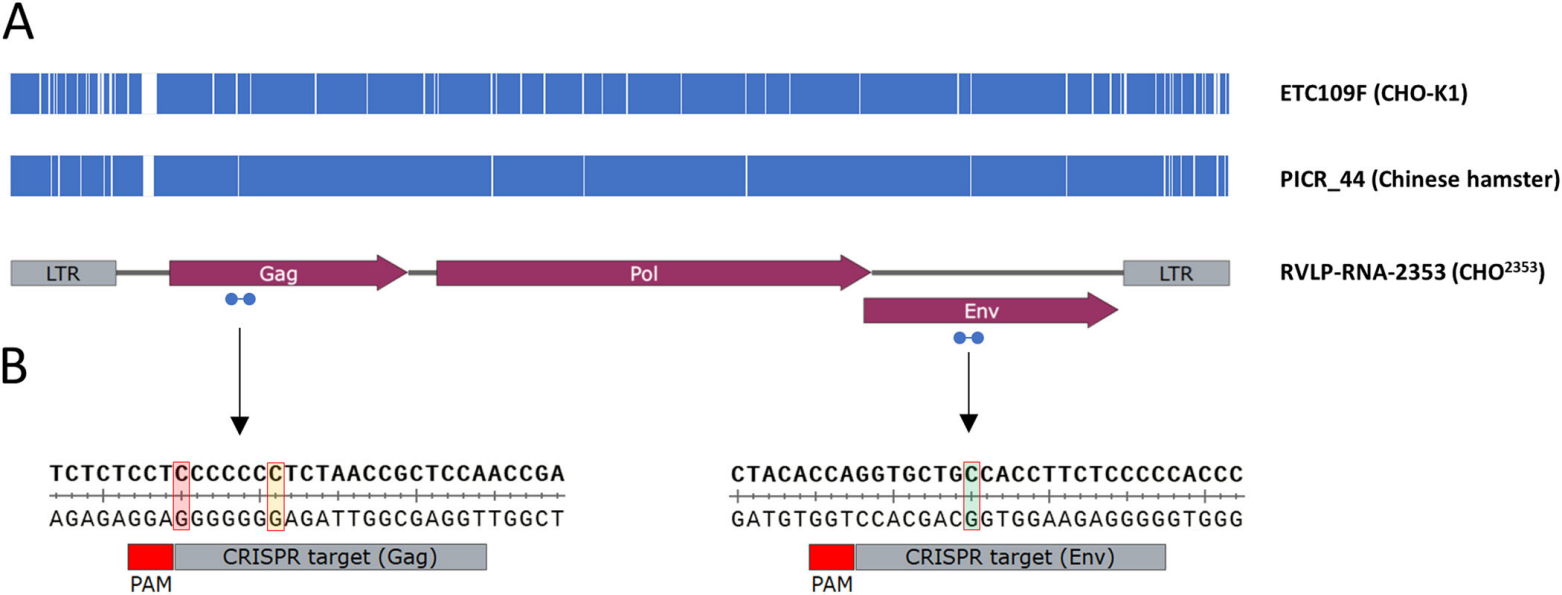
Characterization and CRISPR targeting strategy for endogenous RVLP sequences expressed in CHO^2353^ cells. (A) Features of the predominant endogenous RVLP RNA transcribed in CHO^2353^ cells and comparison to related proviruses detected previously in CHO‐K1 and Chinese hamster genomes. The CHO^2353^ RVLP sequence, determined by sequencing RT‐PCR amplicons from total cellular RNA, contains intact reading frames for type C retrovirus‐like Gag, Pol, and Env proteins. The most similar provirus sequence in the Chinese hamster genome (picr_44, Rupp et al. 2018) contains sequence variants, the locations of which are indicated by vertical white bars. ETC109F, the provirus discovered by Duroy et al. to be responsible for RVLP formation in CHO‐K1 cells, contains additional sequence difference relative to the RVLP sequences expressed in CHO^2353^. (B) Selection of protospacer sequences for CRISPR targeting of RVLP Gag and Env sequences using spCas9. Other RVLP proviruses sequenced from CHO‐K1 or Chinese hamster cells contain mismatches in the selected target sequences: the locations of sequence differences in the Gag sequence in ETC109F and picr_44 proviruses are shown in red and yellow. In the Env target sequence, both ETC109F and picr_44 have a sequence difference at the location shaded in green.

For CRISPR targeting of RVLPs, CHO^2353^ cells were cotransfected using PEI‐Max, as described previously, with plasmids encoding *Streptococcus pyogenes* Cas9 (fused to GFP) and sgRNAs (Koyuturk et al. 2022). For the current study, the sgRNAs contained protospacer sequences corresponding to the CHO^2353^ RVLP Gag or Env sequences shown in Figure 1B.

### 2.6 Design and Cloning of Short Hairpin (sh) RNAs

shRNAs targeting the Pol and Env regions of the RVLP genome were designed using InvivoGen's siRNA Wizard software (https://www.invivogen.com/sirnawizard/). The shRNA loop sequence (CTCGAG) was chosen based on a previous report (Scarborough et al. 2014). Based on InvivoGen readouts, we selected shRNAs targeting Pol (shPol) and Env (shEnv), along with a nonsense shRNA (shNS) (Table 1). All shRNAs in this study were expressed under the U6 promoter, as shRNAs transcribed by this promoter have been shown to persist longer within cells during deep latency in in vitro HIV experiments (Goguen et al. 2021). U6 promoter‐shRNA cassettes were synthesized by Twist Bioscience (San Francisco, USA) and subcloned into the pTT82 plasmid (Poulain et al. 2017) between the SpeI and BglII restriction sites, resulting in removal of the CMV5 promoter and polyadenylation signal. Plasmids were transformed and amplified in *Escherichia coli* DH5α (Invitrogen) using CircleGrow medium (MP Biomedicals) supplemented with 100 µg/mL ampicillin (Gibco). Plasmid purification was performed using an in‐house anion exchange chromatography method, followed by isopropanol precipitation, and a 70% ethanol wash. DNA was quantified using a DeNovix DS‐11+ spectrophotometer. U6 promoter‐shRNA cassette sequences were confirmed by Sanger sequencing using an ABI 3500xl genetic analyzer.

**Table 1:** Sequences of shRNAs designed to target RVLP transcripts in CHO cells.

Name	Sequence
shPol	5′‐GTAAGAGGCTCACCGTGTATAGCTCGAGGTATACACGGTGAGCCTCTTACTTTTTT‐3′
shEnv	5′‐GGCCTTAGTCTTAACTCAACATCTCGAGTTGTTGAGTTAAGACTAAGGCCTTTTTT‐3′
shNS	5′‐GCACCATTCACCCGACAGATTACTCGAGAAATCTGTCGGGTGAATGGTGCTTTTTT‐3′

Abbreviations: CHO, Chinese hamster ovary; RVLP, retrovirus‐like particle.

### 2.7 Transient and Stable shRNA Transfections

Transient shRNA transfections were performed based on (Stuible et al. 2021) with some modifications. Briefly, CHO^2353^ cells at a density of 2 × 10⁶ cells/mL and a viability greater than 98% underwent a complete media exchange before transfection. Dimethylacetamide was added to a final concentration of 0.075% (v/v), and cells were incubated at 37°C, 5% CO₂, and 120 rpm for at least 1 h. Transfections were performed at a final plasmid DNA concentration of 1 µg/mL consisting of 85% (w/w) pTT82‐shRNA, 10% Bcl‐XL plasmid (antiapoptotic effector), and 5% GFP plasmid. Polyplexes were prepared by diluting DNA and PEI‐Max separately in a volume of media equivalent to 1/20 of the final transfected culture volume; diluted PEI‐Max was added to the diluted DNA and incubated at room temperature for 7 min. The DNA/PEI‐Max polyplexes were then added to the cells and incubated for 4 days (96 h). Cell lysates were prepared and analyzed by western blot analysis as described above.

Stable transfection of shRNA plasmids was performed as follows. On the day of transfection, 2 × 10⁶ cells/mL with viability greater than 98% were subjected to complete media exchange before transfection and incubated at 37°C, 5% CO₂, and 120 rpm. Polyplexes were formed as above using pTT82‐shRNA plasmids (with no additional plasmids cotransfected) and added to the cells. Stable transfectants were selected using G418. Twenty‐four hours post‐transfection, viable cell density and viability were assessed, and cells were pelleted and resuspended at 0.5 × 10⁶ cells/mL in selection media consisting of PowerCHO‐2 (Lonza), supplemented with 400 µg/mL G418 (Wisent) and 0.125% anti‐clumping agent A (Sartorius). Cells were returned to incubate under the same conditions, and this process was repeated every 2 to 3 days (seeding cells at 0.5 × 10⁶ cells/mL) with fresh selection media until viability recovered to 90%, at which point cells were diluted (rather than pelleted/resuspended) in selection media to 0.3 × 10⁶ cells/mL every 2–3 days. Cell viability recovered to ≥ 98% within 4 weeks. After this period, the selection media is replaced with maintenance media consisting of BalanCD CHO Growth A (Fujifilm, Irvine Scientific), supplemented with 4 mM l‐glutamine and 0.125% anti‐clumping supplement and cells were cryopreserved.

### 2.8 Flow Cytometry Cell Sorting and Analysis

For Env staining after CRISPR or shRNA transfection, approximately 8 × 10^6^ transiently (5 days post‐transfection with Cas9 and gRNAs) or stably (following G418 selection of cells transfected with shRNA constructs) transfected cells were transferred to 5‐mL Eppendorf tubes, pelleted at 300*g* (5 min) and washed once with cold wash buffer (DPBS + 0.5% BSA + 2 mM EDTA). Cells were pelleted again, resuspended in wash buffer containing 5 µg/mL anti‐Env (5585) and incubated on ice for 30 min. Cells were washed again (as above), resuspended in wash buffer containing AF647‐conjugated anti‐mouse IgG (A31571; Thermo), and incubated for 30 min in the dark on ice. Finally, cells were washed again and resuspended in 2 mL wash buffer for sorting. Cell sorting was performed on a MoFlo Astrios EQ (Beckman Coulter) with debris and multiple cell‐containing droplets excluded based on light scattering signals (forward and side scatter). Cells selected for sorting into 384‐well plates were AF647‐negative (shRNA transfections) or AF647‐negative and GFP‐positive (CRISPR transfection) based on comparison with stained but non‐transfected CHO^2353^ cells.

For evaluation of cell‐surface Env expression in sorted clones expanded to 96‐well plates, the staining protocol was identical, except the procedure was performed in 96‐well V‐bottom plates with reduced buffer volumes (200 µL of wash buffer was used for the wash and final resuspension steps, and primary and secondary antibody staining steps were performed in volumes of 50 and 100 µL, respectively).

### 2.9 CHO Clone Generation

At 10–14 days after single cell sorting into 384‐well plates, plates were scanned using an automated microscope (ImageXpress Micro XLR; Molecular Devices) to assess outgrowth and confluency. Selected clones were transferred to 96‐well plates and cell‐surface Env expression was re‐analyzed by flow cytometry as described above. Selected Env‐negative clones were further expanded into 6‐well plates and shake flasks.

### 2.10 ddPCR Determination of RNA Genome Copy Number

The ddPCR method to quantify RNA genomes present in RVLPs in CHO supernatants was adapted from a published qPCR‐based method (Hussain et al. 2020). To digest RNAs not protected within RVLPs (e.g., RNAs released from dead cells), 500 µL of CHO cell‐free supernatants prepared by low‐speed centrifugation (300*g*, 5 min, unfiltered) were supplemented with 5 µL RNAse I and 56 µL of TNE buffer 10× (Lucigen) and incubated at 37 C for 30 min. The RNAse I was then inactivated by adding 5 µL of 100 mM DTT (for a final concentration of 5 mM) and heating at 70°C for 20 min. RVLP‐associated RNA was then purified using the RNeasy kit (Qiagen), which includes lysis (with RLT Plus buffer) and DNA removal (gDNA Eliminator column) steps, following the manufacturer's instructions. Further removal of contaminating DNA was performed using the optional on‐column DNase incubation included in the Qiagen protocol.

Reverse transcription was performed with 20 ng purified RNA using the Quantitect Reverse Transcription kit (Qiagen), following the manufacturer's instructions (including gDNA Wipeout step). A control reaction (‐RT) was performed to confirm the absence of contaminating RVLP DNA. ddPCR reactions were set up using 5 µL of cDNA or control ‐RT (undiluted or diluted with water to obtain a maximum of 50% positive droplets), 0.1 µM of forward and reverse primers (sequences described previously (de Wit et al. 2000) and QX200 ddPCR EvaGreen Supermix (Bio‐Rad) in a total volume of 25 µL. Droplets were formed in the Bio‐Rad QX200 Droplet Generator using 20 µL of the PCR mix and 70 µL of Droplet Generation Oil for EvaGreen (Bio‐Rad). Thermal cycling was performed using the following conditions: 95°C 5 min (enzyme activation), 35× (95°C 30 s, 60°C 60 s, 72°C 30 s), 4°C 5 min, 90°C 5 min, 12°C hold. Positive and negative droplet counts were determined using the QX200 Droplet Reader (Bio‐Rad), and copy number estimates were calculated using QuantaSoft software (Bio‐Rad).

### 2.11 Spike eVLP Production, Purification, and Analysis

Spike eVLPs were produced by transient transfection of full‐length SARS‐CoV‐2 spike protein, purified from cell‐free supernatants on iodixanol cushions or by spike‐affinity chromatography and analyzed by western blot analysis and transmission electron microscopy (TEM) exactly as described (Alpuche‐Lazcano et al. 2023).

## 3 Results

### 3.1 Characterization of Transcribed RVLP RNAs in CHO‐DXB11‐Derived Clones

When the current project was initiated, apart from the single, nonfunctional, full‐length RVLP sequence published by Lie et al. (ML2G, Genbank accession #U01904) (Lie et al. 1994), little was known about what other RVLP proviruses were present in the CHO genome and which of these could be actively transcribed or encode functional proteins. The Chinese hamster PICR genome, published in 2018 (Rupp et al. 2018), was the first Chinese hamster or CHO cell assembly to be constructed using long‐read PacBio technology in addition to the Illumina‐based methods used for previous assemblies, allowing the locations and sequences of long, repetitive elements like RVLP proviruses to be documented. As it seemed likely that RVLP proviruses related to those expressed by CHO cells would also be present in the animals from which they were derived, we initially searched the PICR assembly for sequences with homology to ML2G. Surprisingly, dozens of sequences with homology to the full length of ML2G (including LTRs) were identified, with many containing intact open reading frames for Gag, Pol, and Env proteins. Despite some differences among these sequences (particularly in the LTRs), to evaluate expression of these sequences in CHO cells, it was possible to design RT‐PCR primer sets targeting internal sequences conserved among the identified collection of potentially functional proviruses (i.e., those in which open reading frames were intact). Using RNA from CHO^55E1^ cells (CHO^55E1^, also named CHO^BRI/rcTA^ is a CHO‐DXB11‐derived clone; Poulain et al. 2017), transcribed proviral sequences were detectable by RT‐PCR (Figure S1A); notably, control PCRs without reverse transcription were also performed and resulted in much lower or undetectable levels of amplified products. This confirms that most of the PCR products are derived from RNA/cDNA rather than proviral sequences in CHO genomic DNA which is a potential contaminant of total RNA preparations.

The RT‐PCR products were subsequently cloned into a plasmid vector and multiple clones derived from each primer set were sequenced. Remarkably, despite the sequence diversity of proviruses at the DNA level in the PICR assembly, most of the transcribed sequences amplified by RT‐PCR had identical sequences. Following this initial experiment, additional primer sets were designed (based on our results and the PICR sequences) to repeat the RT‐PCR/cloning/sequencing workflow and sequence the full length of the RVLP sequences expressed in NRC's CHO‐DXB11‐derived cell lines. Figure S1B shows a schematic of the consensus sequence, RVLP‐RNA‐2353, that was generated from CHO^2353^ cells (a clone generated independently from CHO^55E1^ using the same CHO‐DXB11 parental cells; Joubert et al. 2023), including alignments with all individual sequencing results. Again, there was minimal diversity in the sequences of the different RT‐PCR amplicons: apart from regions of low‐quality sequence (mostly at beginnings and ends of the Sanger sequencing reads) there were only two examples where among all sequences covering a given position, there were single sequences with *bona fide* differences compared to the consensus (both single‐nucleotide changes, indicated in Figure S1B). These polymorphisms may be due to differences in the provirus sequences being transcribed or errors introduced by PCR.

While these analyses were being performed, the sequence of the provirus responsible for RVLP production in CHO‐K1 cells (ETC109F) was published (Duroy et al. 2020). As shown in Figure 1, there are dozens of sequence differences between ETC109F and RVLP‐RNA‐2353. Of note, several matching sequence differences are duplicated in the two LTRs, indicating that these differences existed at the time these proviruses integrated in the CHO (or Chinese hamster) genome, and that they are not simply mutations that have accumulated at the same locus in the time since CHO‐K1 and CHO^2353^ were derived from a common parental cell line. Together, these results strongly suggest that RVLP RNAs are transcribed from distinct proviruses in the CHO‐K1 and CHO^2353^ genomes. The low sequence diversity of transcribed sequences indicates that only one or a few proviruses are likely actively transcribed in CHO^2353^ cells, similar to CHO‐K1 (Duroy et al. 2020). In the Chinese hamster PICR genome (Rupp et al. 2018), the RVLP sequence most similar to RVLP‐RNA‐2353 is found in the PICR_44 contig (we will refer to this as the PICR_44 provirus). As shown in Figure 1A, RVLP‐RNA‐2353 is more similar to PICR_44 than ETC109F. However, several sequence differences in the CHO‐K1‐derived ETC109F (compared to CHO DXB11‐derived RVLP‐RNA‐2353) are shared by PICR‐44, suggesting that ETC109F may be derived from PICR_44 or a very similar ancestral sequence, whereas RVLP‐RNA‐2353 is more distantly related. Nonetheless, despite these polymorphisms, the viral proteins encoded by the three proviruses are extremely similar: comparing RVLP‐RNA‐2353 and ETC109F, the nucleotide sequence differences would translate to a total of only 7 amino acid differences along the entire length of the Gag, Pol, and Env proteins.

### 3.2 CRISPR Targeting of RVLPs in CHO^2353^ Cells

Having established the sequence of a potentially functional RVLP RNA expressed by CHO^2353^ cells, we next attempted to generate RVLP‐deficient CHO cell lines by targeting the associated proviral element(s) in the CHO genome using CRISPR. As shown in Figure 1B, based on our sequencing results, we identified two potential CRISPR target sites (protospacer/PAM sequences), located in the Gag and Env open reading frames, with distinct sequences in RVLP‐RNA‐2353 compared to ETC109F and PICR_44. These sequences are not unique (there are 1–3 matching sequences found in a BLAST search of the PICR assembly with the Gag and Env sequences), but we expected that targeting these less conserved sequences would improve the likelihood of targeting the actively transcribed provirus and reduce the risk of toxicity associated with targeting multiple sites simultaneously using CRISPR, an issue addressed previously during targeting of endogenous retroviruses in pig cells (Yang et al. 2015).

Plasmids were generated that encode CRISPR sgRNAs, under control of human U6 promoter, with targeting sequences (crRNAs) corresponding to these two sites in the Gag and Env sequences. In an initial experiment, these were transfected individually along with a second plasmid encoding spCas9 fused with GFP. Post‐transfection, single GFP+ cells were cloned by sorting into 384‐well plates; after expansion, clones were screened by extracting total cellular RNA and performing RT‐PCR with primer sets encompassing the CRISPR target sites. Figure S2A shows an example of agarose gel electrophoresis of RT‐PCR products generated from Gag‐targeted clones using with the corresponding primer set. Bulk RT‐PCR products were analyzed by Sanger sequencing to identify clones with potential CRISPR‐induced mutations, with example chromatograms and alignments with the reference RVLP‐RNA‐2353 sequence shown in Figure S2B. For clones showing potential mutations, the PCR products were cloned into a plasmid vector so that individual mutated sequences could be determined. For both the Env and Gag CRISPR target sites, we identified several clones with one or two mutated forms of the RVLP‐RNA‐2353 RNA. As examples, RNA from Gag CRISPR clone 25 (CHO^C25^) expresses RNAs containing 4‐ or 8‐bp deletions at the Gag target site (Figure S2C) while Env CRISPR clone 9 (CHO^C9^) shows 10‐ or 13‐bp deletions at the Env target site (Figure S2D). Importantly, although a limited number of PCR products were cloned and sequenced, non‐mutated RVLP‐RNA‐2353 RNAs were no longer detected in CHO^C25^ or CHO^C9^, suggesting that only two genome‐integrated copies of the RVLP‐RNA‐2353 provirus are being expressed by the parental CHO^2353^ cells and that these were successfully targeted using our CRISPR strategy. Also, the lengths of the deleted sequences in these clones would interrupt the reading frames of the corresponding Gag and Env genes, very likely disrupting the functions of any translated proteins.

### 3.3 Development of Antibody Reagents for Detection of CHO RVLP Proteins

To date, there are no commercially available antibodies against CHO RVLP proteins or even any published literature in which development of such antibodies has been reported. Given the potential applications for CRISPR KO clone screening and characterization as well as for monitoring the presence of RVLPs during recombinant protein purification processes, we sought to develop a panel of monoclonal antibodies (mAbs) against the CHO RVLP Gag and Env proteins. Mice were immunized and mAb‐secreting hybridomas were isolated using standard methods, and hybridoma supernatants were initially screened for binding to RVLP Env and Gag by ELISA (not shown).

Budding type‐C endogenous RVLPs from CHO cells correspond to the *Gammaretrovirus* genus, and RVLP‐RNA‐2353, ETC109F, and PICR_44 all show significant sequence similarity to the murine leukemia virus (MLV) and feline leukemia virus (FeLV) (Duroy et al. 2020). The well‐characterized MLV Gag polyprotein (p65) is processed into different functional domains, including p15 (matrix), p30 (capsid), p10 (nucleocapsid), and p12 (Leis et al. 1988). Notably, intermediately‐processed forms of retrovirus Gag can be detectable, as demonstrated for human immunodeficiency virus (HIV) (Coren et al. 2007).

Anti‐Gag mAbs were next tested by western blot analysis using cell‐free CHO^2353^ culture supernatants. As shown in Figure S3A, approximately half of the mAbs positive for binding by ELISA detect a dominant protein band at ~30 kDa with additional less abundant higher‐molecular‐weight species by western blot analysis. The immunogen we used for Gag antibody development was based on the predicted RVLP p30 sequence, so this is very likely the dominant band detected by western blot analysis with CHO^2353^ supernatants. The higher‐molecular‐weight species detected by the anti‐RVLP‐Gag mAbs likely correspond to incompletely‐processed forms of the polyprotein. In later experiments, we confirmed for certain mAbs (e.g., 5660) that the detected proteins are indeed different forms of Gag, as they are not detected in lysates or supernatants of CRISPR‐induced KO cells.

For the anti‐Env mAbs, the western blot testing was performed with lysates of CHO^2353^ and Env‐KO CHO^C9^ cells. As shown in Figure S3B, several mAbs show strong binding to a protein of ~80 kDa, which corresponds to the expected molecular weight of CHO RVLP Env protein (amino acid sequence corresponds to a molecular weight of 69 kDa but 7 potential N‐glycosylation sites are present). This protein is further confirmed to be Env as the full‐length band is not detected with lysates from CHO^C9^ cells. Interestingly, mAbs 5845 and 5847 detect a lower molecular weight band in CHO^C9^ cell lysates which is not detected by mAbs 5638 or 5590. This band likely represents a truncated form of Env resulting from the CRISPR‐induced mutations in CHO^C9^ and indicates that mAbs 5845/5847 are not binding the same epitope as 5638/5590.

We also tested whether the anti‐Env mAbs can detect endogenous RVLP Env protein on the surface of live CHO^2353^ cells by flow cytometry. As shown in Figure S3C, the fluorescence signal after staining with most anti‐Env mAbs was higher for wild‐type CHO^2353^ cells compared to Env‐KO CHO^C9^, and the signal was particularly strong for some mAbs (e.g., 5585, 5638 and 5590), with little overlap between fluorescence histograms of positive (CHO^2353^) and negative (CHO^C9^) cells. This robust resolution of Env‐positive and ‐negative populations suggested that that flow cytometry‐based cell sorting could be used to enrich Env‐knockout cells from bulk CRISPR‐transfected cell populations.

### 3.4 Env Antibody Enrichment for RVLP‐Deficient CHO Cell Line Development

**Figure 2:**
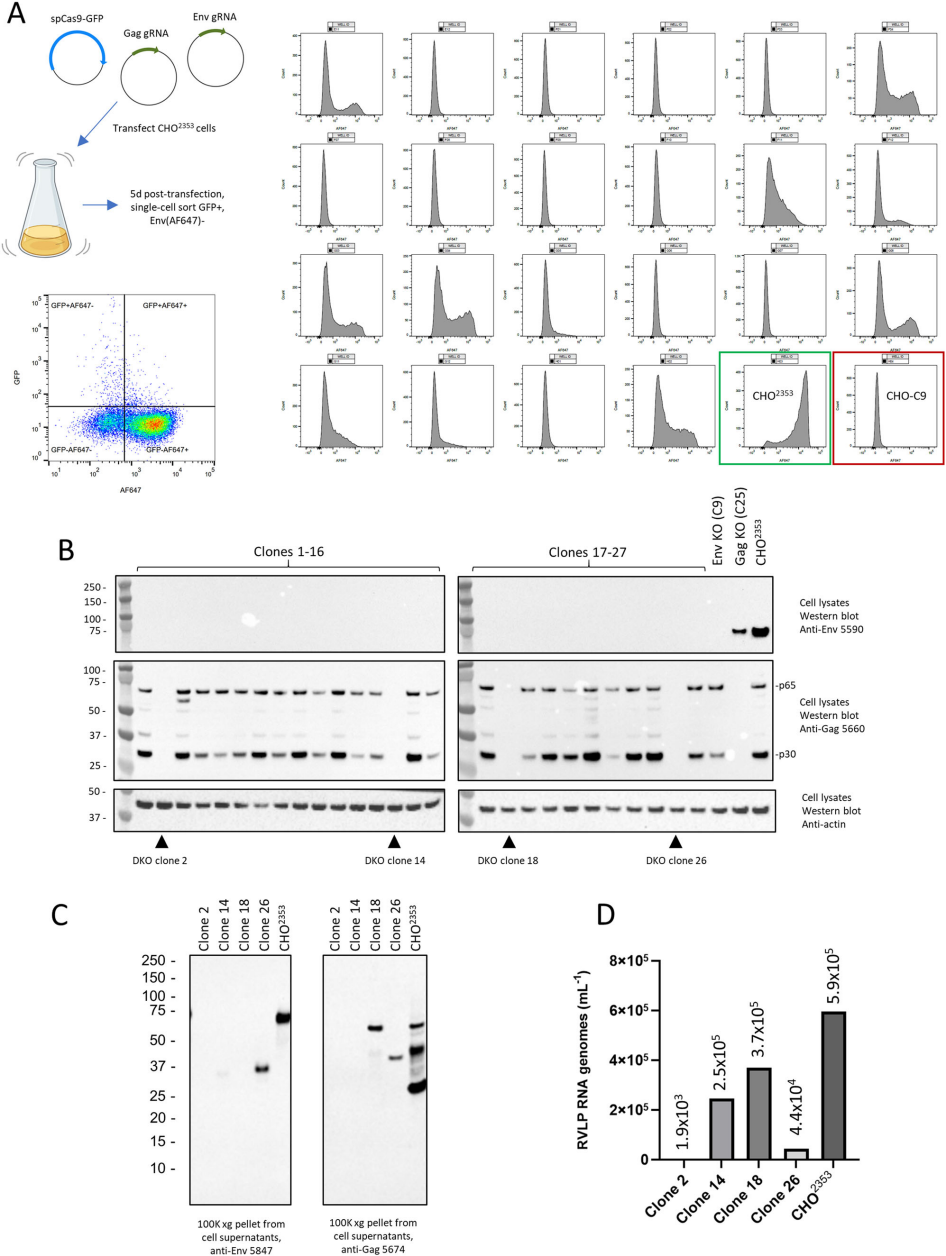
Identification of RVLP Gag/Env KO clones is facilitated by enrichment of bulk CRISPR‐targeted cells for those lacking cell‐surface Env expression by flow cytometry. (A) CHO^2353^ cells were cotransfected with plasmids encoding Cas9‐GFP and gRNAs targeting RVLP Gag and Env sequences. Five days post‐transfection, single GFP+, Env (AF647)‐negative cells (top left quadrant of dot plot) were single‐cell‐sorted into 384‐well plates. After expansion into 96‐well plates, clones were screened for Env expression by FACS. Selected Alexa Fluor 647 fluorescence histograms are shown in the right panel for candidate knockout clones as well as control CHO^2353^ (Env‐positive, green box) and CHO^C9^ (Env‐negative, red box) cells. (B) Protein lysates, prepared from Env‐negative clones identified by flow cytometry, were analyzed by western blot analysis for Env and Gag protein expression. (C) High‐speed centrifugation pellets from culture supernatants from Gag/Env double knockout clones were analyzed by western blot analysis for Gag and Env expression. (D) RVLP RNA genome copy numbers in culture supernatants of double‐knockout and control CHO^2353^ cells were analyzed by ddPCR.

After the initial success targeting RVLP Gag or Env individually, we used CRISPR again to attempt to generate Env/Gag double‐knockout CHO cells, this time taking advantage of the new antibodies to facilitate FACS enrichment and clone screening. Parental CHO^2353^ cells were cotransfected with plasmids encoding spCas9‐GFP and the same Gag and Env sequence‐specific gRNAs used previously. At 5 days post‐transfection, cells were stained with anti‐Env mAb 5585 (binding detected with a secondary anti‐mouse AF647‐labelled antibody). Single GFP+/Env− cells were sorted into 384‐well plates. Clones were expanded and screened for Env cell‐surface expression by flow cytometry. As shown in Figure 2A, the majority of clone‐derived cell lines consisted of predominantly Env‐ cells, with a few showing a mix of positive and negative cells. We selected 27 clones for further expansion and screening by western blot analysis. As shown in Figure 2B, all 27 clones that were identified as negative by flow cytometry were also negative for Env protein expression in cell lysates by western blot analysis. Anti‐Gag western blot analysis with the same lysate revealed 4 potential double‐knockout clones, clones 2, 14, 18, and 26, that were also negative for Gag protein, including the predominant full‐length (p65) and processed (p30) forms. To verify that the reduction in Env and Gag protein in cell lysates from these clones corresponded to reduced RVLP production, we performed high‐speed centrifugation of conditioned media (media collected 8 days post‐seeding of different cell lines at 1 × 10^6^/mL) and analyzed sedimented material, which should contain RVLPs, by western blot. As expected, full‐length Env and different processed forms of Gag were detectable in pellets derived from parental CHO^2353^ cell supernatants (Figure 2C). All four putative double‐knockout clones show reduced levels of Env and Gag in sedimented conditioned media. However, residual full‐length or truncated Gag and/or Env proteins are still detectable for clones 14, 28, and 26, whereas both are undetectable for clone 2. A digital droplet RT‐PCR (ddRT‐PCR) assay was developed to quantify RVLP genomic RNA in CHO cell supernatants, based on previously published qPCR‐based methods (Hussain et al. 2020; de Wit et al. 2000); importantly, this method includes an RNAse I digestion to eliminate RNA not protected within intact RVLP particles as well as a DNAse digestion to remove contaminating CHO cell genomic DNA (and associated RVLP proviral DNA). As shown in Figure 2D, while RVLP genomic RNA is reduced in clones 14, 18, and 26 compared to CHO^2353^ cells, it was clone 2 (CHO^C2^) that produced the lowest levels of RVLP RNA. For parental CHO^2353^ cells, a control performed without reverse transcriptase enzyme gave at least 1000× lower copy number estimates, demonstrating that there is minimal genomic DNA contamination (data not shown).

**Figure 3:**
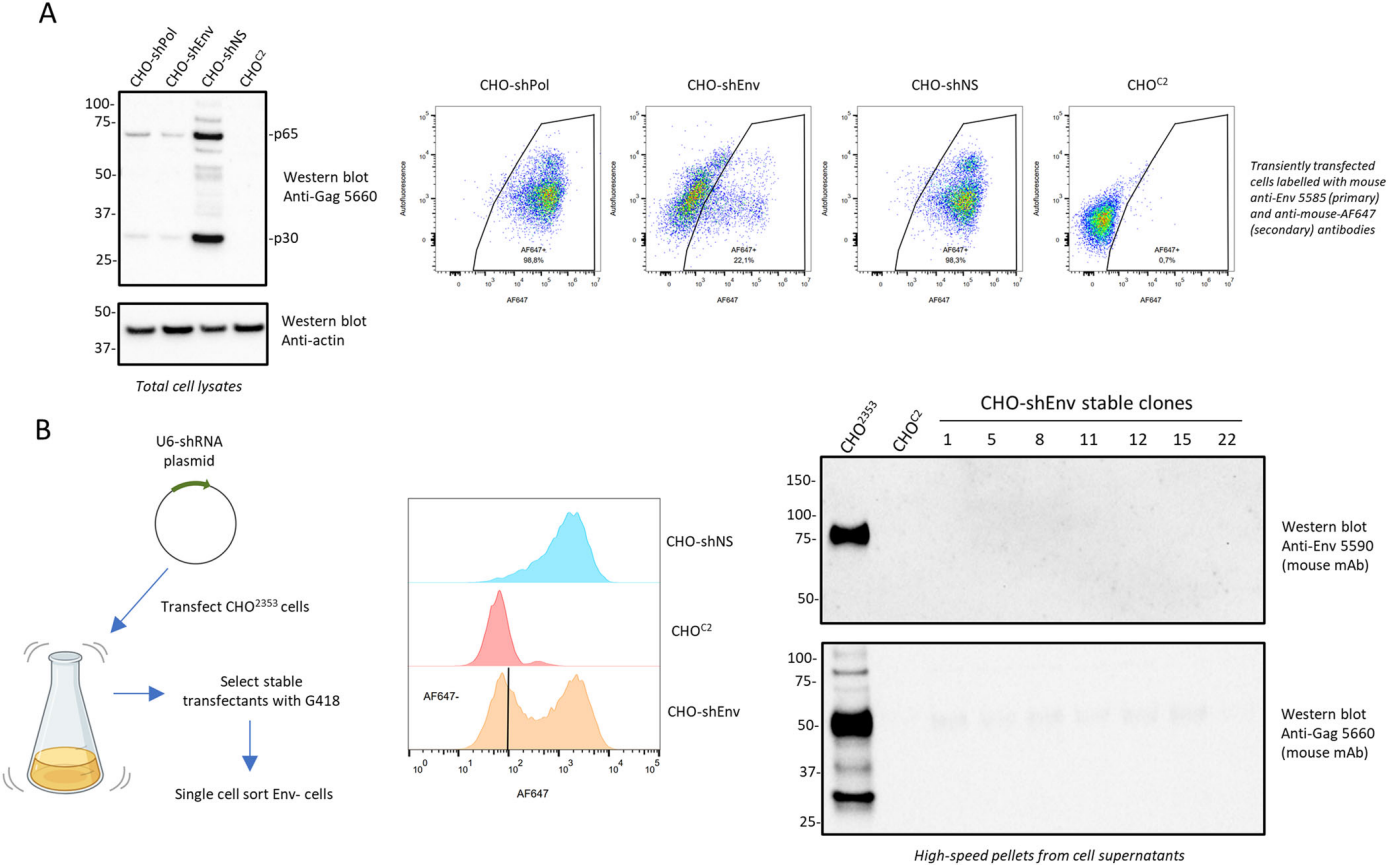
Short hairpin RNAs (shRNAs) can effectively down‐regulate RVLP protein expression in CHO cells, and enriching shRNA‐induced Env‐negative cells by FACS can be used to identify stable RVLP‐knockdown cells. (A) Downregulation of RVLP protein expression by transient transfection of shRNA plasmids. CHO^2353^ cells were transiently transfected with plasmids encoding shRNAs against RVLP Pol (shPol) or Env (shEnv) or a non‐sense sequence (shNS). At 96 h post‐transection, cell lysates were prepared for western blot analysis (left panel) or stained with anti‐RVLP Env (mAb 5585) and analyzed by flow cytometry (right panel). (B) Generation of RVLP‐knockdown CHO cells by stable expression of shEnv. Stably transfected CHO^2353^‐derived pools expressing shEnv or shNS were analyzed by flow cytometry following cell‐surface Env staining and compared to control CRISPR‐knockout (CHO^C2^) cells (middle panel). The indicated Env (AF647)‐negative population of the shEnv‐transfected pool was selected for single‐cell cloning. After expansion, cell supernatants of stable clones were analyzed by western blot analysis (cell supernatants were pelleted by ultracentrifugation to concentrate RVLP particles).

As an alternative strategy to engineer RVLP‐deficient cells, we tested transfection of plasmid‐encoded shRNAs targeting RVLP sequences. As shown in Figure 3A, we initially evaluated Gag and Env expression by western blot and flow cytometry, respectively, following transient transfection of CHO^2353^ cells. Compared to cells transfected with a nontargeting shRNA (shNS), shRNAs targeting sequences within both the Pol and Env genes resulted in reduced expression of all isoforms of the Gag protein (left panel). Transfection of Env‐targeted shRNA resulted in reduced cell‐surface Env expression detected by flow cytometry (right panel). Interestingly, while both Pol‐ and Env‐targeted shRNAs caused reduced Gag expression, only the Env‐targeted shRNA affected Env expression. This can be explained by the splicing mechanism of retroviruses; for instance, murine leukemia virus (MLV) produces two or three distinct transcripts (depending on the variant) from its proviral form: the genomic RNA (8.3 kb), the singly spliced RNA that generates SD′ RNAp50 (4.4 kb), and a fully spliced transcript, SD RNAEnv (3 kb) (Pessel‐Vivares et al. 2015). Notably, both full‐length and spliced transcripts contain Env sequences (Déjardin et al. 2000) making the Env‐targeted shRNA capable of downregulating all expressed transcripts. To achieve more durable reduction of RVLP expression, we isolated stably transfected pools by G418 selection. Env‐negative cells from the shEnv‐transfected pool were sorted using a flow cytometer as performed previously with CRISPR‐targeted cells. High‐speed centrifugation pellets from the conditioned media of expanded clones were analyzed by western blot analysis for Env and Gag expression. As shown in Figure 3B, Env expression was not detectable in this sedimented material in the shEnv‐expressing stable clones; Gag expression was greatly reduced compared to CHO^2353^ cells, but residual protein was still detectable, in particular a partially processed form of ~50 kDa. Together, these results demonstrate that both CRISPR and shRNA‐based approaches can effectively reduce CHO RVLP protein expression and that Env antibody‐based enrichment can be used to enrich stably transfected, non‐clonal cells for knockdown/knockout clones.

### 3.5 Recombinant Enveloped VLP Production by RVLP‐Deficient CHO Cells

**Figure 4:**
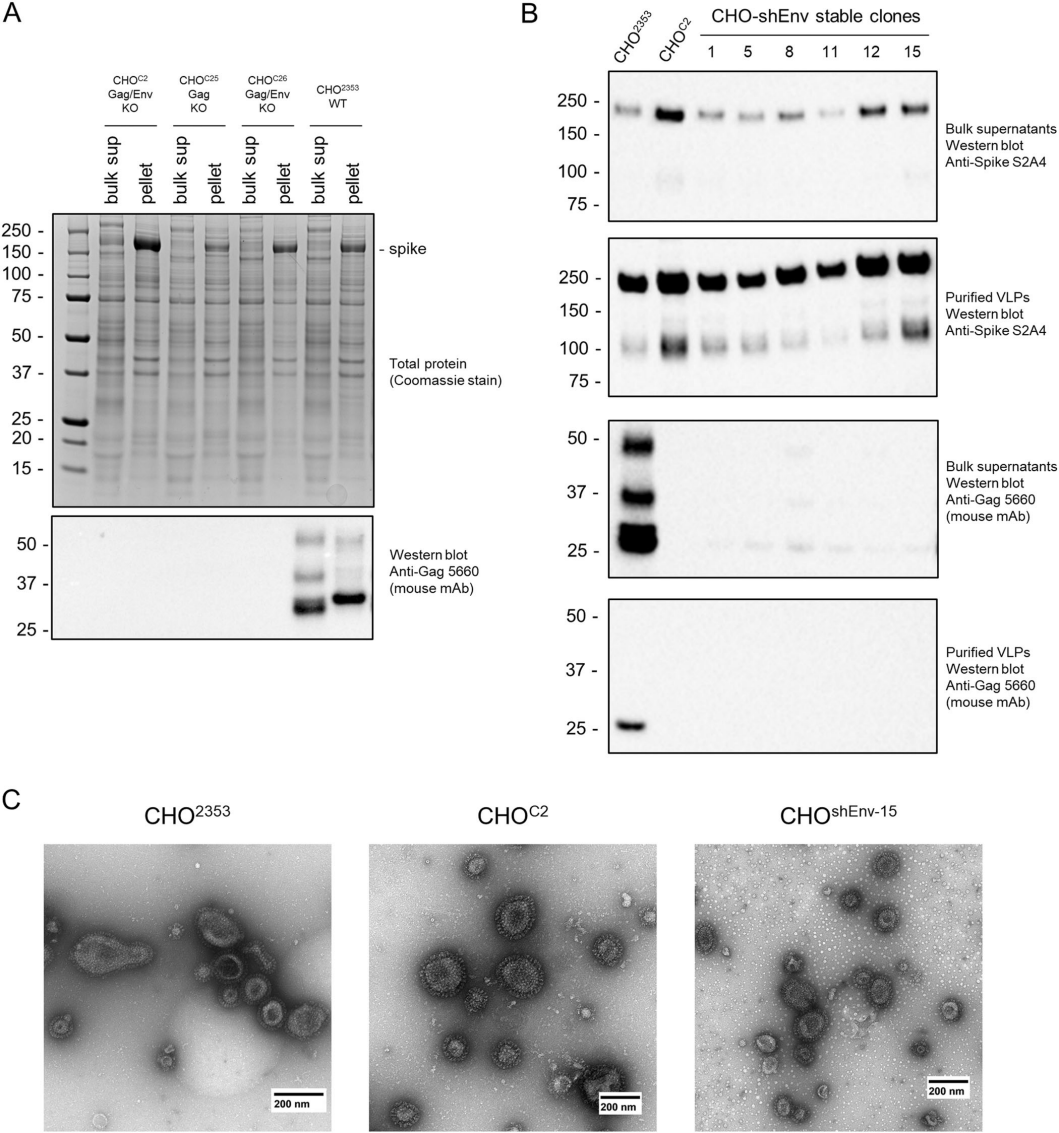
CRISPR‐RVLP‐knockout and shRNA‐RVLP‐knockdown CHO clones produce SARS‐CoV‐2 spike VLPs with yields similar to parental CHO^2353^ cells. (A) Production of spike VLPs by transient transfection of selected CRISPR knockout clones and CHO^2353^ cells. At 5 d post‐transection, bulk supernatants (“bulk sup”) or VLPs purified by sedimentation on an iodixanol cushion (“pellet”) were analyzed by SDS‐PAGE and Coomassie staining or western blot analysis. (B, C) Comparison of spike VLP production by transient transfection of stable Env shRNA (shEnv) clones, CHO^2353^, and CRISPR clone CHO^C2^. At 5 d post‐transfection, supernatants were harvested and VLPs were purified by spike‐affinity chromatography. Bulk supernatants and purified VLPs were analyzed by western blot analysis (B). Purified VLPs were also analyzed by transmission electron microscopy (C).

An important potential application of RVLP‐deficient CHO cells could be for production of recombinant, enveloped VLPs (eVLPs) as therapeutics, in particular as viral vaccine antigens (Sanchez‐Martinez et al. 2024a); eVLPs can have biophysical properties similar to RVLPs, so if RVLP‐producing cells were used to manufacture an eVLP therapeutic, purification and viral clearance/inactivation are expected to be very challenging. We demonstrated recently that SARS‐CoV‐2 spike protein overexpression in CHO cells can induce production of spike‐coated eVLPs (Alpuche‐Lazcano et al. 2023) which have further potential to co‐display influenza virus antigens (Sanchez‐Martinez et al. 2024b). To test the potential of newly‐developed RVLP‐deficient CHO clones for spike eVLP production, we expressed full‐length SARS‐CoV‐2 spike sequence (with intact transmembrane/C‐terminal domains) by transient transfection in CRISPR‐knockout (Figure 4A) or shRNA‐knockdown (Figure 4B) clones and compared eVLP production to parental CHO^2353^ cells. As shown in Figure 4A, spike protein can be observed after total protein (Coomassie Blue) staining of iodixanol cushion‐purified supernatant from CHO^2353^ cells and also for CRISPR knockout clones CHO^C2^, CHO^C26^ (Env/Gag double knockouts), and CHO^C25^ (Gag‐only knockout). There is clone‐to‐clone variability for the knockouts, with CHO^C2^ producing the most VLPs based on spike protein content. Notably, RVLP Gag protein is detected, as expected, in the CHO^2353^ bulk (unpurified) supernatant and iodixanol fractions (iodixanol purifies particles based on density and should not discriminate between spike VLPs and RVLPs), but not in the samples from the knockout clones. For six shRNA knockdown clones, the same transient spike VLP productions were performed alongside CHO^2353^ and CHO^C2^ cells as controls. In this case, VLPs were purified by spike affinity chromatography rather than iodixanol. As shown in Figure 4B, all shRNA clones produced similar or higher levels of VLPs than CHO^2353^ cells, but none produced more than the CHO^C2^ CRISPR clone. Interestingly, despite being purified by affinity chromatography, RVLP Gag protein is still detected by western blot in VLPs from CHO^2353^ cells, highlighting the potential of RVLP proteins to contaminate eVLP preparations, even when a high‐specificity purification process is applied. Finally, as shown in Figure 4C, TEM analysis indicates that the morphologies of affinity‐purified eVLPs produced by wild‐type CHO^2353^ and RVLP‐deficient CHO^C2^ and CHO^shEnv15^ cell lines are similar. Together, these results demonstrate that RVLP‐deficient CHO cells have potential to produce similar or higher levels of recombinant VLPs than unmodified RVLP‐producing cells.

## 4 Discussion

The presence of RVLPs in CHO cell supernatants was recognized in the early 1970s (Lieber et al. 1973), while CHO cells were first used as a production host for therapeutic protein manufacturing in the late 1980s (Collen and Lijnen 2009). Since then, coinciding with an explosion in approvals of monoclonal antibodies and other protein‐based biologics derived from CHO cells, the biomanufacturing industry has established effective procedures for quantifying RVLPs combined with downstream purification (DSP) processes that can achieve sufficient viral inactivation/clearance to ensure product safety (Ajayi et al. 2022). Nonetheless, the presence of RVLPs still contributes to the complexity and cost of therapeutic protein manufacturing: for example, dedicated chromatography, low‐pH or detergent‐based viral inactivation, and viral filtration steps may be necessary during DSP primarily for the purpose of reducing RVLP levels. Furthermore, these viral clearance/inactivation steps may be incompatible with some proteins and more complex biologics such as enveloped VLPs. These factors underly the potential utility of engineered RVLP‐deficient cell lines for biomanufacturing.

Our current study builds on the findings of Duroy et al. published in 2020, who demonstrated that despite an abundance of proviral sequences in their genome, RVLP production by CHO‐K1 cells could be effectively prevented by CRISPR‐mediated mutation of just a single integrated provirus (Duroy et al. 2020). In our CHO‐DXB11‐derived cell line (CHO^2353^), we sequenced the predominant RVLP RNA expressed by these cells and implemented a similar CRISPR targeting strategy. While Duroy et al. targeted conserved regions of the RVLP Gag sequence, we instead selected CRISPR target sites within both Gag and Env that have sequence polymorphisms in the sequenced RNA compared to most or all genome‐integrated proviruses in the Chinese hamster PICR genome (Rupp et al. 2018), with the intention of improving targeting specificity. Like the earlier paper, generation of CRISPR knockouts was successful, for both Gag and Env sites targeted individually or in combination.

We did not perform an extensive transcriptomic or genomic analysis of the parental or CRISPR‐targeted cells as conducted by Duroy et al. However, our other results strongly suggest that, similar to CHO‐K1 cells, a very small set of genome‐integrated proviruses are responsible for RVLP production by CHO^2353^ cells. First, for RVLP RNAs amplified by RT‐PCR and analyzed by Sanger sequencing, there was very low sequence diversity: in the Chinese hamster PICR assembly, different RVLP proviruses have signature sequence polymorphisms, but for a given primer set, the great majority of CHO^2353^‐derived RT‐PCR amplicons all shared the same sequence. This indicates that the RNAs are not the products of transcription of a large number of different proviruses. Likewise, for the single Gag‐ or Env‐knockouts generated by CRISPR, we identified a maximum of two targeted knockout alleles expressed as mutated RNAs in each cell line. Given the random nature of the DNA repair process that generates indels at sites of CRISPR cleavage, if a larger number of proviruses were contributing to RVLP RNA expression, we would have expected the knockout cells to express RVLP RNAs with more diverse CRISPR‐induced mutations. Although we cannot reach a solid conclusion without comparing the full genome sequence of parental and knockout cells, our results suggest that RVLP production by CHO^2353^ is the result of two independent integrated proviruses with highly similar or identical sequences. Notably, RVLPs from CHO‐K1 cells appear to be the product of one, rather than two proviruses, which is highly similar but not identical to the sequence expressed in CHO^2353^ cells; given the pattern of sequence differences and comparison to other proviruses in the PICR genome, it is likely that RVLPs are being expressed from different proviral loci in these two cell lines. Interestingly, CHO‐DXB11 cells (from which CHO^2353^ were derived) were derived from CHO‐K1 in 1980 (Urlaub and Chasin 1980)—we can speculate that since then, the activated/silenced state of different proviruses has changed and/or new integration events have occurred which have led to changes in the dominance of different proviral elements.

CRISPR/Cas9 is an exceptionally powerful tool for cell engineering, and in the current study, we have confirmed that it can be used to disrupt endogenous RVLP production by CHO cells, even in the context of a genome harboring hundreds of highly similar proviral sequences. Nonetheless, there are some drawbacks to using CRISPR for engineering CHO cells to be used for commercial manufacturing. In particular, the intellectual property landscape surrounding commercial use of CRISPR‐modified producer cell lines is unclear (Storz 2024). For this reason, we believe that the shRNA knockdown approach validated in the current study could be particularly impactful. The original patents for use of shRNAs to control gene expression in mammalian cells are expired (Fire et al. 2003), so intellectual property concerns should not limit commercial development of shRNA‐expressing cells. Furthermore, the U6 promoter‐shRNA cassette required for shRNA knockdown of RVLPs is very small, so unlike Cas9, it could potentially be integrated in transgene‐encoding plasmids for stable, constitutive expression without needing to re‐engineer industry‐standard parental cell lines or modify existing cell line development workflows. We have not formally assessed the stability of the RVLP‐deficient phenotype of CRISPR‐ or shRNA‐targeted cells. We envision different factors potentially impacting the stability of the two approaches: for CRISPR, although particular proviruses can be genetically modified to render them nonfunctional, a large number of non‐expressed RVLP proviral elements exist in the CHO genome, and it is possible they could be re‐activated under certain circumstances. For shRNA, if the plasmid encoding the shRNA cassette is integrated in an unstable manner in the CHO genome (as observed occasionally for protein‐encoding plasmids), RVLP knockdown could be lost. The stability of RVLP deficiency should certainly be monitored if such cells are eventually used for biologics manufacturing. Likewise, off‐target effects are possible for both CRISPR and shRNA targeting approaches; in particular, CRISPR can cause DNA breaks and mutations at unintended genomic sites while shRNA expression (specifically from the U6 promoter) can affect expression of cellular RNAs requiring RNA polymerase II for transcription (Grimm et al. 2006), among other potential effects. Such impacts were not investigated in the current study.

A significant advancement made in the current study was the development of antibodies against RVLP Env and Gag proteins and their application for enrichment and characterization of RVLP‐deficient CHO clones. With the Env antibodies, we demonstrated that RVLP Env is readily detectable on the surface of CHO^2353^ cells. This finding prompted the use of the same antibodies to enrich bulk CRISPR‐ and shRNA‐transfected CHO cell pools for Env‐deficient cells. Using CRISPR to disrupt Gag or Env individually without any such enrichment step, we found that ~10% of derived clones contained CRISPR‐induced mutations. For generating double knockouts in a similar manner, we could therefore expect that ~1% of clones would have mutations in both genes. Env antibody enrichment allowed a homogenous Env‐negative population to be obtained by flow cytometry‐based cell sorting, from which Gag‐negative double knockout cells could be much more efficiently identified. A similar strategy was used to select Env‐knockdown clones expressing Env‐targeted shRNA, with our shRNA design causing concomitant reduction in Gag expression in the Env‐knockdown cells. Beyond cell enrichment, Env and Gag antibodies were used in our study to confirm the reduction in RVLP protein levels in knockdown/knockout cell lysates and supernatants by western blot analysis. We think that these antibody reagents have potential for a variety of additional applications including ELISAs, flow virometry, and other immune techniques to facilitate detection and quantification of RVLPs in CHO cell harvests and during DSP.

A detailed discussion of how RVLP‐deficient CHO cell lines would be accepted by regulators is beyond the scope of this manuscript; however, the stringency of viral clearance required by regulators during DSP of a given CHO‐derived product is largely dependent on evaluation of RVLP concentrations in unprocessed bulk harvest material from bioreactors, using a validated method (usually transmission electron microscopy) (Ajayi et al. 2022). If such testing would confirm the reductions detected by the ddPCR method in the current study, we expect that, at least from a viral clearance perspective, a less stringent DSP process may be acceptable. As already discussed, additional testing to verify stability of RVLP knockdown/knockout as well as evaluation of off‐target effects of CRISPR/Cas9 would likely also be important for regulatory approval.

The current study, along with that of Duroy et al. (2020). demonstrate that using modern cell engineering techniques, it can be quite straightforward to generate RVLP‐deficient CHO cell lines. So far, we have generated and characterized a limited number of such cell lines and have not observed any consistent impacts of RVLP deficiency on cell growth rates or cell densities reached during shake‐flask fed‐batch productions (data not shown); therefore, although it has not been tested, we do not anticipate issues with future scale‐up to bioreactors for manufacturing. Given the well‐established procedures, refined over decades, currently used by the biomanufacturing industry to remove and/or inactivate RVLPs during DSP, there is understandably some reluctance to change the strategy used to mitigate potential risks posed by these particles. We also note that procedures used to address the presence of RVLPs have potential to reduce risk of some adventitious agents as well. However, in our opinion, there are significant advantages to focusing efforts on eliminating the root cause of RVLPs, that is, the presence of transcribed proviruses in CHO cell genomes, rather than only on how to remove/inactivate them post‐production. We believe that there is great potential to facilitate not only manufacturing of conventional recombinant protein therapeutics (e.g., monoclonal antibodies) but also expand the limits of CHO cell “factories” to produce more complex biologics such as VLPs (e.g., Alpuche‐Lazcano et al. 2023; Sanchez‐Martinez et al. 2024a, 2024b) and viral vectors (e.g., Nagy et al. 2023; Cao et al. 2024) which would not be compatible with current RVLP mitigation procedures.

## Author Contributions

**Matthew Stuible:** original draft preparation (lead); review and editing (equal); conceptualization (equal); investigation (equal); supervision (equal). **Sergio P. Alpuche‐Lazcano:** review and editing (equal); conceptualization (equal); investigation (equal). **Christian Gervais:** conceptualization (equal); investigation (equal). **Manon Ouimet:** investigation (equal). **Julie Lippens:** investigation (equal). **Martine Pagé:** investigation (equal). **Audrey Morasse:** investigation (equal). **Anna N. Moraitis:** supervision (equal). **Yves Durocher:** review and editing (equal); conceptualization (equal); supervision (equal).

## Conflicts of Interest

The authors declare no conflicts of interest.

## Supporting information

RVLP manuscript supplementary figures.

## Data Availability

The data that support the findings of this study are available from the corresponding author upon reasonable request.
