# Supplementary material for: Endogenous Retrovirus‐Like Particle‐Deficient CHO Cells Can be Generated by CRISPR or shRNA and Enriched Based on Cell‐Surface Expression of Retroviral Envelope Protein
Source: Biotechnol Bioeng. 2025 Aug 22;122(11):3192–204. doi: 10.1002/bit.70043 (PMC12503009; doi:10.1002/bit.70043)
Supplement: Supplementary file 1 — RVLP manuscript supplementary figures. [file BIT-122-3192-s001.pdf]

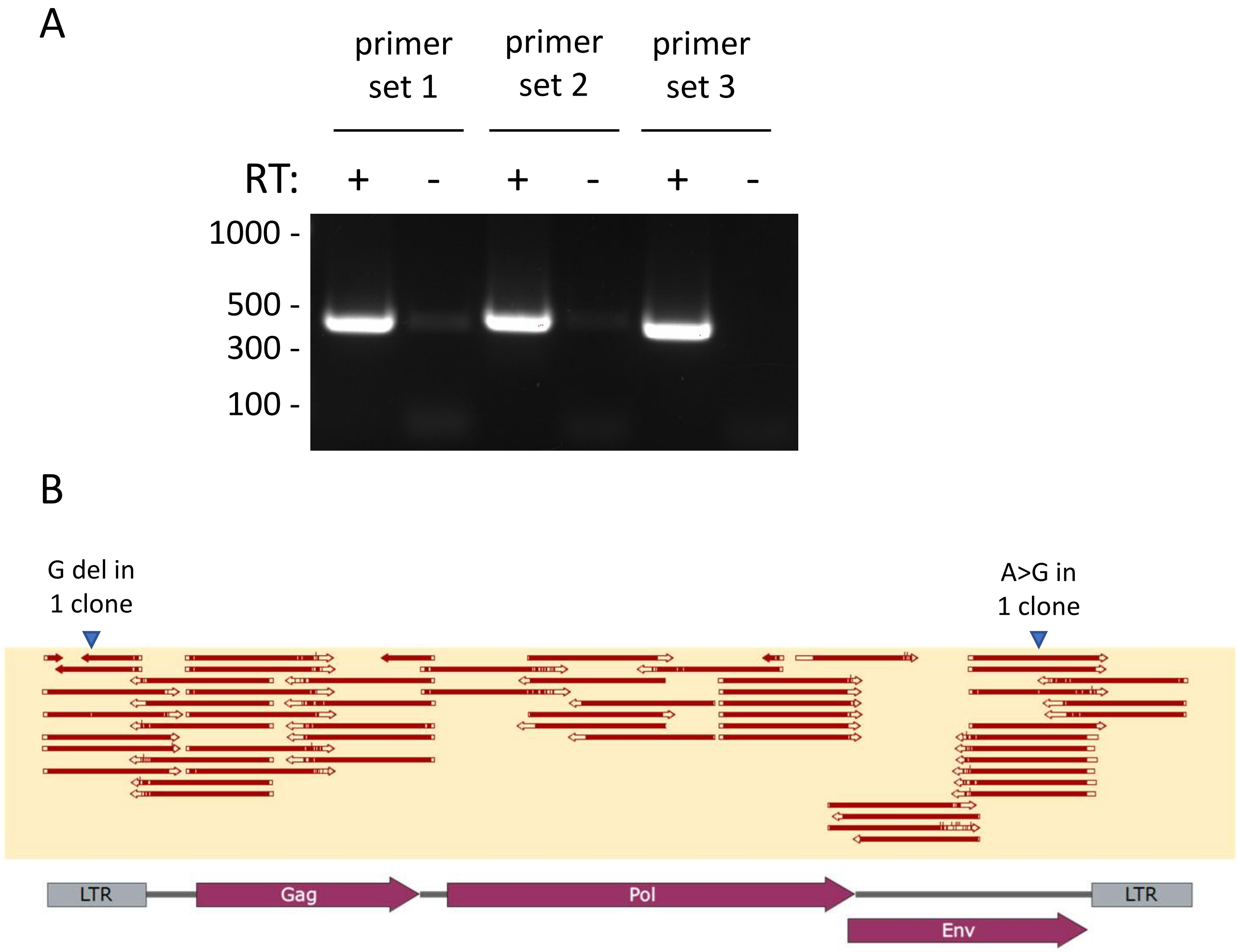

Supplementary Figure 1: RVLP RNAs detected in CHO-DXB11-derived cell lines show low sequence diversity. (A) cDNA was prepared by reverse transcription (RT) of total cellular RNA from CHO^{55E1} cells. A control reaction was performed without RT. RT-PCR was performed with cDNA (+RT) and control (-RT) templates using primer sets 1 (ATAACACTGCAAGTCGGGGG, GGCCCATTCCTCCTGTTTCA), 2 (ATCCCCTACCGGGTAACCAA, AACAAGGTGGCCACTGACTG) or 3 (TGGGCAACTACTCCAACCAG, AATTCCCTGTAGGGCAACGG), and products were analyzed by agarose gel electrophoresis. (B) RT-PCR products generated as described in (A) with additional primer sets using RNA from CHO^{2353} cells were cloned and sequenced by Sanger sequencing. Individual sequencing results are aligned with a consensus sequence above a schematic showing locations of RVLP Gag, Pol and Env genes and long terminal repeats (LTRs). Alignments shown in dark red color are identical to the consensus.

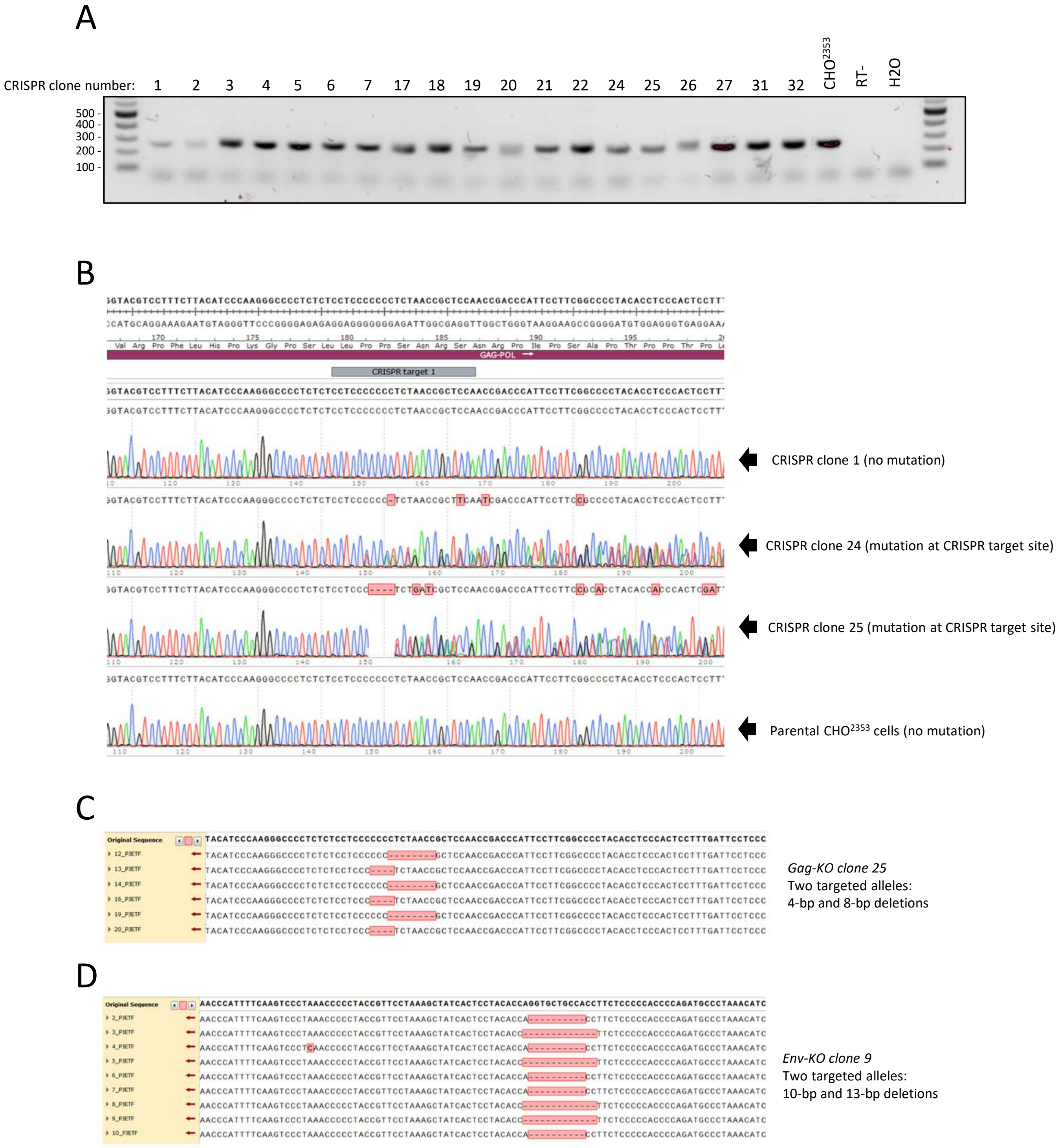

Supplementary Figure 2: CRISPR clone screening workflow for identification of single Gag and Env-knockout CHO clones. (A) Agarose gel of products of RT-PCR reactions using total cellular RNA from CHO clones following CRISPR targeting with gRNA specific for the RVLP Gag sequence. PCR primers flanked the CRISPR target site (B) Sanger sequencing of bulk RT-PCR products to identify potential clones with CRISPR-induced mutations. (C) Sequencing of RT-PCR products for Gag CRISPR clone 25 following cloning in cloneJet plasmid. (D) Sequencing of RT-PCR products for Env CRISPR clone 9 following cloning in cloneJet plasmid.

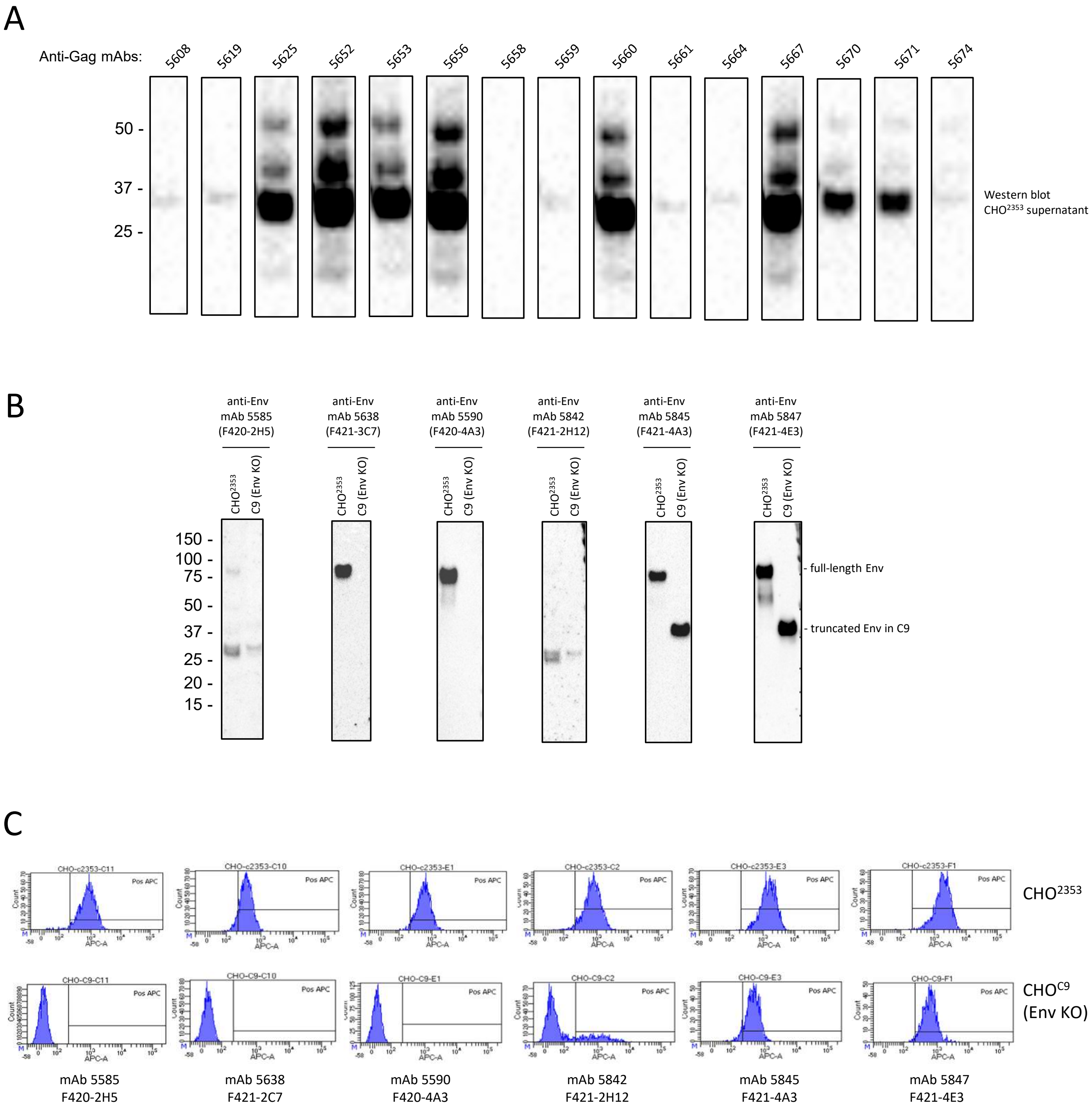

Supplementary Figure 3: Validation of RVLP Gag and Env antibodies for flow cytometry and western blotting. (A) RVLP Gag mAbs were tested by western blotting using supernatants from CHO^{2353} cells. RVLP Env mAbs were tested for detection of Env in lysates of CHO^{2353} or Env-KO CHO-C9 cells by western blotting (B) or on the surface of live cells by flow cytometry (C).
